# Evidence of Brain Alterations in Noncerebral Falciparum Malaria

**DOI:** 10.1093/cid/ciab907

**Published:** 2021-12-15

**Authors:** Sanjib Mohanty, Praveen K Sahu, Rajyabardhan Pattnaik, Megharay Majhi, Sameer Maharana, Jabamani Bage, Akshaya Mohanty, Anita Mohanty, Martin Bendszus, Catriona Patterson, Himanshu Gupta, Arjen M Dondorp, Lukas Pirpamer, Angelika Hoffmann, Samuel C Wassmer

**Affiliations:** Center for the Study of Complex Malaria in India, Ispat General Hospital, Rourkela, Odisha, India; Center for the Study of Complex Malaria in India, Ispat General Hospital, Rourkela, Odisha, India; Department of Intensive Care, Ispat General Hospital, Rourkela, Odisha, India; Department of Radiology, Ispat General Hospital, Rourkela, Odisha, India; Center for the Study of Complex Malaria in India, Ispat General Hospital, Rourkela, Odisha, India; Center for the Study of Complex Malaria in India, Ispat General Hospital, Rourkela, Odisha, India; Infectious Diseases Biology Unit, Institute of Life Sciences, Bhubaneswar, Odisha, India; Department of Intensive Care, Ispat General Hospital, Rourkela, Odisha, India; Department of Neuroradiology, University Hospital Heidelberg, Heidelberg, Germany; Department of Infection Biology, London School of Hygiene & Tropical Medicine, London, United Kingdom; Department of Infection Biology, London School of Hygiene & Tropical Medicine, London, United Kingdom; Mahidol Oxford Tropical Medicine Research Unit, Faculty of Tropical Medicine, Mahidol University, Bangkok, Thailand; Centre for Tropical Medicine & Global Health, Nuffield Department of Clinical Medicine, Oxford, United Kingdom; Department of Infection Biology, London School of Hygiene & Tropical Medicine, London, United Kingdom; Department of Neuroradiology, University Hospital Heidelberg, Heidelberg, Germany; University Institute of Diagnostic and Interventional Neuroradiology, University Hospital Bern, Inselspital, University of Bern, Bern, Switzerland; Department of Infection Biology, London School of Hygiene & Tropical Medicine, London, United Kingdom

**Keywords:** acute kidney injury, Brain, brain-kidney cross-talk, cytotoxic edema, MRI, *Plasmodium falciparum* infection, S100B, Severe malaria, vasogenic edema

## Abstract

**Background:**

Cerebral malaria in adults is associated with brain hypoxic changes on magnetic resonance (MR) images and has a high fatality rate. Findings of neuroimaging studies suggest that brain involvement also occurs in patients with uncomplicated malaria (UM) or severe noncerebral malaria (SNCM) without coma, but such features were never rigorously characterized.

**Methods:**

Twenty patients with UM and 21 with SNCM underwent MR imaging on admission and 44–72 hours later, as well as plasma analysis. Apparent diffusion coefficient (ADC) maps were generated, with values from 5 healthy individuals serving as controls.

**Results:**

Patients with SNCM had a wide spectrum of cerebral ADC values, including both decreased and increased values compared with controls. Patients with low ADC values, indicating cytotoxic edema, showed hypoxic patterns similar to cerebral malaria despite the absence of deep coma. Conversely, high ADC values, indicative of mild vasogenic edema, were observed in both patients with SNCM and patients with UM. Brain involvement was confirmed by elevated circulating levels of S100B. Creatinine was negatively correlated with ADC in SNCM, suggesting an association between acute kidney injury and cytotoxic brain changes.

**Conclusions:**

Brain involvement is common in adults with SNCM and a subgroup of hospitalized patients with UM, which warrants closer neurological follow-up. Increased creatinine in SNCM may render the brain more susceptible to cytotoxic edema.

## Teaser

Quantitative brain magnetic resonance imaging analyses of cerebral tissue water diffusion showed brain changes in adults with severe noncerebral malaria and uncomplicated malaria, suggesting that *Plasmodium falciparum*infection often affects this organ, despite the absence of coma.

*Plasmodium falciparum* infections accounted for an estimated 229 million clinical cases in 2019, resulting in 409000 deaths [1]. Severe falciparum malaria is a life-threatening, multiorgan disease with a variety of clinical presentations, including cerebral malaria (CM), metabolic acidosis, hyperparasitemia, acute kidney injury (AKI), hepatic dysfunction, severe anemia, and hypoglycemia [2]. The pathogenesis of coma in CM is still incompletely understood, but microvascular impairment in the brain by sequestration of infected erythrocytes (IEs) is a central feature [3]. Inflammation of brain parenchyma is minimal, although intravascular leukocytes are more prominent in African children than in Asian adults who died of CM [4, 5]. There is a mild generalized increase in systemic vascular permeability, and imaging studies show only limited cerebral swelling in most adult patients, whereas cerebral edema is more prominent in African children, particularly in the agonal stages [6–9].

Over time, the World Health Organization–defined criteria for CM have broadened, allowing the identification of a substantial additional group of patients with mild cerebral involvement and emphasizing that neurological involvement in severe malaria occurs on a gradual scale [10–13]. Our team previously reported the common presence of mild cerebral changes on magnetic resonance (MR) imaging in Indian patients with uncomplicated malaria (UM), suggesting that falciparum malaria infection affects the brain in the absence of coma [14]. A separate study from Bangladesh also described brain parenchymal changes on MR images of adult patients with severe malaria and without neurological symptoms presenting [15]. AKI, liver failure, and anemia are common in adults with severe falciparum malaria [2, 16] and may independently contribute to these brain changes [17–19].

Here, we provide a first comprehensive assessment of brain MR imaging in adult patients with severe noncerebral malaria (SNCM) [13]. We performed a systematic quantitative assessment of apparent diffusion coefficient (ADC) maps, using MR imaging with diffusion-weighted imaging to allow the discrimination between cytotoxic and vasogenic edema. This approach was complemented by measurement of parasite biomass and plasma S100B, a marker of brain injury, as well as correlation analyses between ADC values and laboratory parameters associated with AKI, hepatic dysfunction, and severe anemia, to assess their potential impact on brain changes observed in SNCM.

## PATIENTS AND METHODS

### Study Site

The study was carried out at Ispat General Hospital in Rourkela, India. Written consent was obtained from all enrolled subjects before inclusion in the study. Ethical approvals are listed in the Supplementary Material.

### Patients and Controls

Adult patients with parasitologically proven UM or SNCM were enrolled in the study, using criteria described in the Supplementary Material. A group of 5 healthy adults underwent MR imaging of the brain and served as a control group.

### MR Imaging and Analysis

Brain MR imaging was performed using a 1.5-T Siemens Symphony MR imager (Siemens). The degree of brain swelling was assessed on T2-weighted images and graded according to sulcal effacement and cortical swelling (Supplementary Material). Our group previously showed that subtle ADC alterations in patient with UM can be revealed by quantitative analyses of ADC values [14]. ADC map–derived whole-brain histograms were generated and used for differentiation between cytotoxic and vasogenic edema [14]. Normalized whole-brain ADC histograms were created, and the peak location of those histograms corresponding to the most common ADC value in the brain tissue [20] was used for the analyses. The range of ADC values from 5 healthy controls was used as baseline.

### Plasma Levels of S100B and *P. falciparum* Histidine-Rich Protein 2

Plasma levels of S100B, a widely validated peripheral biomarker of blood-brain barrier permeability and central nervous system injury [21], and *P. falciparum* histidine-rich protein 2 (PfHRP2), an indicator of the total parasite biomass [22], were assessed (Supplementary Material).

### Statistical Analyses

We used χ^2^ tests to compare categorical variables. Depending on the normality distribution, unpaired Student *t* or Mann-Whitney tests was used to compare 2 groups. Pearson correlation coefficients were calculated for correlation analyses. Differences were considered statistically significant at *P* < .05 (2 sided). All statistical analyses were performed using GraphPad Prism 8.3 (GraphPad Software).

## RESULTS

### Baseline Characteristics

Between October 2013 and November 2019, 21 adult patients with SNCM, 20 patients with UM, and 5 healthy control subjects were enrolled in the study. Baseline characteristics are summarized in Table 1. Two patients with SNCM had Glasgow Coma Scale (GCS) scores of 12 and 14, and the rest had a GCS score of 15. All patients survived. Brain imaging was carried out within 10 hours of admission. Follow-up MR imaging of the brain after a mean (standard deviation [SD]) of 52.95 (14.22) hours was feasible in 34 of 41 patients (83%).

**Table 1.** Clinical and Laboratory Parameters in Patients With Uncomplicated or Severe Noncerebral Malaria

Parameter	Patients With UM (n = 20)	Patients With SNCM (n = 21)
Demographics		
Age, mean (SD), y	40 (14)	38 (14)
Sex, no. female; no. male	6; 14	3; 18
Parasite burden		
Parasitemia, no.	20	21
Parasitemia, median (IQR), ×10^3^ parasites/μL	2.31 (0.54–72.70)	4.72 (0.31–199.45)
PfHRP2, no.	10	20
PfHRP2, median (IQR), ng/mL	48.64 (8.55–640.75)	281.57 (21.73–960.43)
Clinical parameters, mean (SD)		
Platelet count	61.23 (38.11)	46.95 (58.57)
Hemoglobin, g/dL	10.50 (2.27)	8.63 (3.38)
Bilirubin, mg/dL	1.44 (0.68)	7.19 (5.28)
Creatinine, mg/dL	1.05 (0.27)	2.17 (1.75)
S100B, pg/mL	826.2 (1027)	9213 (11335)
GCS score	14.95 (0.22)	14.86 (0.48)
Other parameters		
Follow-up images, no.	15	19
Follow-up time, mean (SD), h	50.37 (48.14–71.22)	47.38 (44.45–50.29)

Abbreviations: GCS, Glasgow Coma Scale; IQR, interquartile range; PfHRP2, *Plasmodium falciparum* histidine-rich protein 2; SD, standard deviation; SNCM, severe noncerebral malaria; UM, uncomplicated malaria.

### Qualitative MR Findings in SNCM

**Figure 1.**
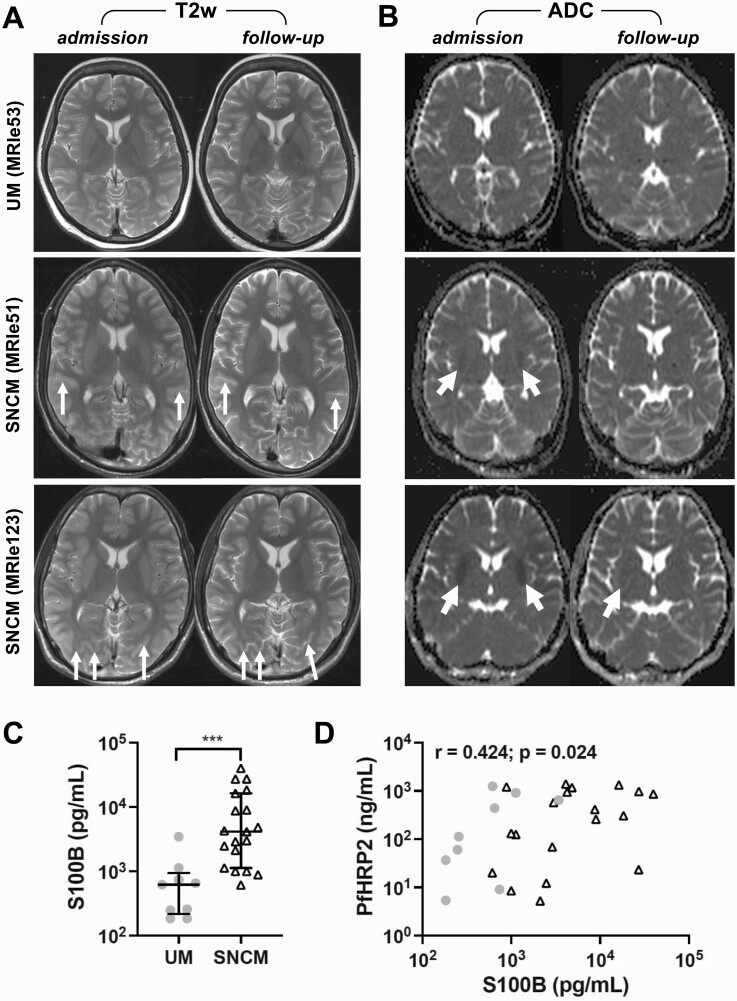
Qualitative assessment of apparent diffusion coefficient (ADC) alterations and brain swelling. *A, B,* Representative T2-weighted (T2w) images (*A*) and ADC maps (*B*) in 1 patient with uncomplicated malaria (UM) and 2 with severe noncerebral malaria (SNCM) at admission (*left-hand images*) and at follow-up (*right-hand images*). *Top row of images,* Patient with UM (patient MRIe53) shows neither visual brain swelling (*A*) nor ADC alterations (*B*). *Middle row,* In 1 patient with SNCM (patient MRIe51), mild brain swelling with sulci narrowing is visible (*A, left-hand image* [*arrows*]) and has normalized at follow-up imaging (*A, right-hand image* [*arrows*]). In the same patient, a slight ADC decrease is visible at admission (*B, left-hand image* [*arrows*]), which has resolved at follow-up (*B, right-hand image*). *Bottom row,* In contrast, patient MRIe123 had mild brain swelling evidenced by sulci narrowing at admission (*A, left-hand image* [*arrows*]), which had normalized at follow-up (*A, right-hand image* [*arrows*]); ADC maps in this patient were comparable to those seen in cerebral malaria [14]: a clear ADC decrease is evident in the basal ganglia at admission (*B, left-hand image* [*arrows*]), with residual changes at follow-up (*B, right-hand image* [*arrow*]). *C,* Plasma levels of S100B in patients with UM or SNCM, shown as medians with interquartile range. ^∗∗∗^*P* < .005. *D,* S100B concentrations are plotted against plasma levels of *Plasmodium falciparum* histidine-rich protein 2 (PfHRP2) in patients with UM or SNCM.

Mild brain swelling was previously reported on MR images of adult patients with SNCM, and a high signal on diffusion-weighted imaging was seen in 1 patient [15]. In contrast, healthy brains showed well-delineated outer cerebrospinal fluid spaces with no signs of volume increase in adjacent brain structures. In this study we observed mild brain swelling on T2-weighted images in 5 of 21 patients with SNCM (24%), without evident signal increase in brain structures (Figure 1A). We quantified diffusion-weighted imaging in patients with SNCM and generated associated ADC maps. In healthy brains, all structures had similar ADC values without visually evident increase or decrease. An ADC decrease in the basal ganglia was observed in 4 of 21 patients with SNCM (19%) (Figure 1B). One of these 4 patients with SNCM had the lowest GCS score (12 of 15) and the strongest ADC alterations in the basal ganglia, accompanied by mild brain swelling. One patient had a small cytotoxic lesion of the corpus callosum (Supplementary Figure 1).

### Elevated Plasma S100B Levels in Patients With SNCM

To confirm brain involvement in SNCM, we measured plasma levels of S100B, which increases in a variety of pathological conditions of the nervous system [21]. Plasma samples were available for only 10 of 20 patients with UM (Table 1). Patients with SNCM had a significantly higher levels of S100B than patients with UM (*P* = .0002; Figure 1C), confirming a degree of brain injury in patients with severe falciparum malaria with or without mildly decreased GCS scores. Plasma levels of S100B, which is degraded by the kidneys, were not correlated with creatinine (Supplementary Figure 2). However, when UM and SNCM patients were combined, these levels were correlated positively with PfHRP2, a marker of parasite biomass (Figure 1D).

### Quantitative ADC Assessment to Identify Patient Subgroups With Cytotoxic or Vasogenic Edema

**Figure 2.**
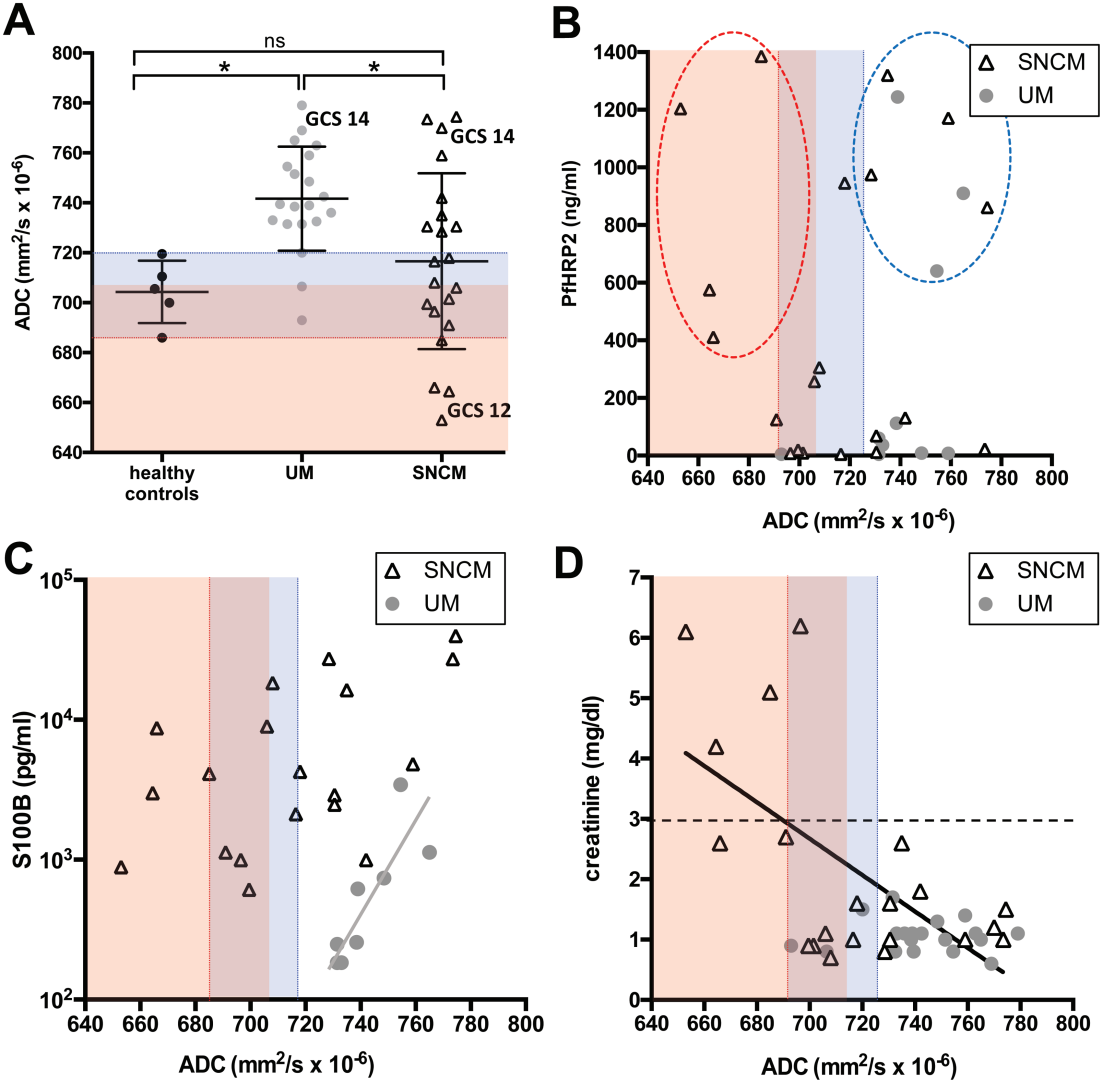
Quantitative apparent diffusion coefficient (ADC) changes and correlation with laboratory values. *A*, Quantitative whole-brain ADC values in healthy controls, patients with uncomplicated malaria (UM), and patients with severe noncerebral malaria (SNCM). Red-shaded area represents the range of ADC values described in patients with cerebral malaria (CM) [14]; blue-shaded area, the range of healthy control values. Patients with Glasgow coma scale (GCS) scores <15 are shown next to the corresponding ADC values. Most patients with UM have ADC values higher than those in healthy controls, while patients with SNCM had both higher and lower ADC values than controls, with values in the latter group similar to those reported in patients with CM. ^∗^*P* < .05; NS, not significant. *B–D,* Correlations between ADC values and levels of *Plasmodium falciparum* histidine-rich protein 2 (PfHRP2), S100B, and creatinine in patients with UM and or SNCM; red lines represent the lowest and the blue lines the highest healthy control ADC values. *B,* High PfHRP2 concentrations were noted in all patients with SNCM with low ADC values (*red-outlined oval*), whereas half of patients with SNCM and one-third of those with UM with high ADC values also had high parasite biomass (*blue-outlined oval*). *C,* In patients with UM, S100B levels were correlated positively with ADC values (*r* = 0.9; *P* = .003). *D,* In patients with SNCM, high creatinine levels were correlated with low ADC values (*r* = 0.43; *P* = .02); black dotted line represents World Health Organization cutoff value for acute kidney injury in *P. falciparum* infection.

We performed analyses of ADC map–derived whole-brain histograms to determine the presence of cytotoxic (low ADC values) or vasogenic (high ADC values) edema (Figure 2A). Patients with SNCM showed a wide spectrum of ADC alterations (mean [SD], 716.6 [35.2] × 10^−6^ mm^2^/s) compared with patients with UM (741.7 [20.8] × 10^−6^ mm^2^/s) and healthy controls (704.3 [12.5] × 10^−6^ mm^2^/s) (Figure 2A). Patients with SNCM had ADC values on admission that were either lower (4 of 21 patients; mean [SD], 667.1 [13.26] × 10^−6^ mm^2^/s), within the same range (8 of 21; 704.6 [9.418] × 10^−6^ mm^2^/s), or higher (9 of 21; 749.3 [19.81] × 10^−6^ mm^2^/s) than in the control group (Figure 2A). In line with the visual qualitative assessments, the patient with SNCM with the lowest GCS score (12 of 15) had the lowest whole-brain ADC value of 653 × 10^−6^ mm^2^/s, in the range of ADC values for adult CM [14]. In contrast, high ADC values in patients with SNCM were in the same range as those in patients with UM (Figure 2A). Most patients with UM (17 of 20 [85%]) also had higher ADC values than healthy controls (mean [SD], 747.9 [14.8] × 10^−6^ mm^2^/s).

### AKI Correlated With Whole-Brain ADC Values in Patients With SNCM

Because parasite sequestration may contribute to cytotoxic edema, we assessed whether parasite biomass, measured by plasma levels of PfHRP2, influenced whole-brain ADC values. There was no linear correlation between ADC values and PfHRP2, but all 4 patients with SNCM with low ADC values (100%) showed higher parasite biomass than patients with normal-range ADC values (Figure 2B). In addition, 4 of 8 patients with SNCM with high ADC values (50%) also had high parasite biomass, indicating that high PfHRP2 may be associated with either low or high ADC values. Three of 9 patients with UM and high ADC values (33%) showed high parasite biomass (Figure 2B). ADC values and S100B levels were strongly correlated in patients with UM (*r *= 0.92; *P* = .003) (Figure 2C).

In patients with SNCM, no correlation was seen between S100B and ADC values, indicating that brain alterations in SNCM are likely multifactorial. To investigate whether ADC changes are potentially driven by noncerebral organ involvement, we correlated ADC values with plasma creatinine, bilirubin, and hemoglobin levels and compared these laboratory measurements between UM and SNCM groups. Plasma creatinine concentrations were significantly increased in SNCM compared with UM, related to the high frequency of AKI in the former group (Supplementary Figure 3*A*). Moreover, creatinine values in patients with SNCM were negatively correlated with whole-brain ADC values (*r*^2^ = 0.37; *P* = .003) (Figure 2D). Bilirubin levels were also significantly higher in patients with SNCM than in those with UM but did not show any correlation with whole-brain ADC values (Supplementary Figure 3*B*). Hemoglobin concentrations did not differ between UM and SNCM and were not related to ADC values (Supplementary Figure 3*C*).

### ADC Values at 72-Hour Follow-up

**Figure 3.**
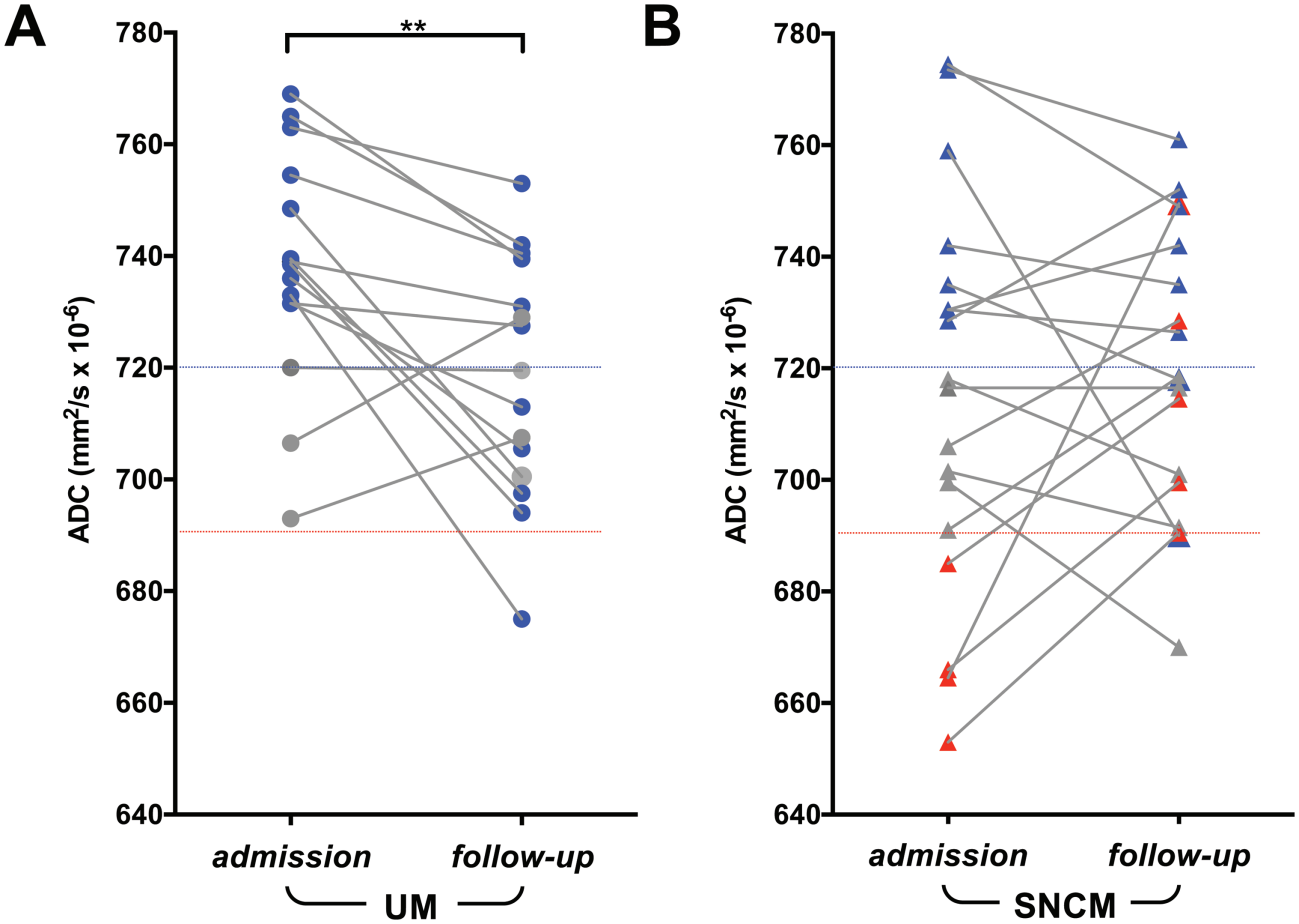
Temporal apparent diffusion coefficient (ADC) changes in patients with uncomplicated malaria (UM) or severe noncerebral malaria (SNCM). ADC values at admission and follow-up are presented. Red line represent the lowest and blue lines the highest healthy control ADC values. Blue symbols represent decreased ADC values at follow-up imaging; gray symbols, no change in ADC values; and red symbols, increased ADC values at follow-up. *A,* Patients with UM show a significant ADC decrease at follow-up compared with admission ADC values. ^∗∗^*P* < .005. *B,* Patients with SNCM show either increased or a decreased ADC values compared with healthy controls.

A significant decrease in ADC values was observed in patients with UM after antimalarial treatment (*P* = .006; mean [SD], 717.3 [21.41] × 10^−6^ mm^2^/s). In 5 patients, these values returned to normal (mean [SD], 704.3 [12.5] × 10^−6^ mm^2^/s), while 6 patients still had values above the healthy range 48–71 hours after admission. One patient with UM had ADC values below and 1 had values above the healthy range at follow-up MR imaging (Figure 3A). Similarly, in SNCM cases, most ADC values showed a reversal trend toward the healthy control range (mean [SD] ADC value in all patients with SNCM at follow-up, 719.6 [25.61] × 10^−6^ mm^2^/s) (Figure 3B).

ADC values increased significantly in all 4 patients with low ADC values at admission (mean value [SD], 713.5 [25.96] × 10^−6^ mm^2^/s; *P* = .04) (Supplementary Figure 4*A*), indicative of reversed cytotoxic edema. One patient had an ADC value above the healthy range on the second image, suggesting a shift from cytotoxic to mild vasogenic edema (Supplementary Figure 4*A*). Patients with SNCM with normal ADC values at admission showed either no change at follow-up (4 of 6 patients), ADC increase toward the range associated with vasogenic edema (1 of 6), or a decrease toward the cytotoxic edema range (1 of 7) (mean [SD], 704.3 [21.39] × 10^−6^ mm^2^/s) (Supplementary Figure 4*B*). Most patients with high ADC values at admission showed a decrease in ADC values at follow-up, but apart from 2 patients, values remained higher than at baseline, indicating an incomplete reversal of vasogenic edema (mean [SD], 734.2 [22.65] × 10^−6^ mm^2^/s; S3C). Two patients showed an increase in ADC values at follow-up (Supplementary Figure 4*C*).

## DISCUSSION

This study used MR imaging with quantitative ADC imaging to assess the presence of vasogenic and cytotoxic edema in patients with falciparum malaria without pronounced clinical neurological involvement, including SNCM and UM. ADC values indicating mild vasogenic edema were observed in a subset of patients with SNCM and UM and were similar to the range observed in UM cases from an earlier study [14]. In contrast, ADC values indicating cytotoxic edema were noted in a subset of patients, with ADC values like those reported in CM [14].

Increased ADC values can result from various conditions, including increased cerebral vascular permeability [23], anemia [24, 25], or liver failure [26]. As we did not see a correlation between hemoglobin or bilirubin levels and ADC values, mild endothelial dysfunction aggravated by a high parasite biomass in a subgroup of patients with SNCM or UM may cause the observed increase in brain ADC values, which is consistent with both the endothelial activation [27, 28] and the sequestration of IEs described in falciparum malaria [3]. In an MR imaging study from Bangladesh of adults with severe malaria, mild brain swelling was noted by visual assessment in up to 50% of patients with SNCM, related at least in part to increased cerebral blood volume caused by high parasite biomass [15]. Using the same approach, we observed a lower rate of mild swelling in our cohort (24%). However, a quantitative assessment of whole-brain ADC values revealed an ADC increase in 43% of patients with SNCM, indicative of mild vasogenic edema and associated with brain swelling.

An earlier computed tomography study from Ispat General Hospital showed that mild to moderate brain swelling was common in patients with severe malaria, both with and without CM [6]. In patients with CM, coma depth was not associated with the severity of brain swelling. At computed tomography, however, the different contributors to brain swelling could not be distinguished. The present study shows that mild vasogenic edema is present in both patients with SNCM and patients with UM, usually normalizing after 72 hours. Neither of these patient groups have decreased GCS scores, suggesting that mild vasogenic cerebral edema does not cause prominent neurological symptoms during acute disease. However, longer-term neurocognitive sequelae were not assessed, and high plasma levels of S100B in both the SNCM group and a subgroup of patients with UM may indicate a degree of permanent damage, as reported in a broad range of diseases [29–31].

In severe malaria, high levels of S100B in the CSF were associated with seizures in both Kenyan children and Vietnamese adults [32, 33]. Acute seizures are a major risk factor for neurological sequelae after recovery in pediatric CM [34]. Our results indicate for the first time that additional studies evaluating the occurrence of long-term neurological sequelae are warranted in SNCM, and possibly in UM.

In 4 of 21 patients with SNCM (19%), ADC values compatible with cytotoxic edema were observed in the same areas of the brain as in patients with CM, including the basal ganglia. This is in line with an earlier study from Bangladesh, where evidence of cytotoxic edema was found in 13% of patients with SNCM [15]. Follow-up imaging showed that in all 4 patients with cytotoxic edema, ADC values normalized after 72 hours, indicating reversibility of the process. In addition to the potential effect of microvascular sequestration of IEs, reversible cytotoxic lesions of the corpus callosum have been associated with increased levels of proinflammatory cytokines and extracellular glutamate [35]. However, it is impossible to identify neuronal cell loss after cytotoxic swelling in the follow-up images, and more permanent neurological damage cannot be excluded in these patients. In 1 patient with SNCM with a GCS score of 15, we noted a small cytotoxic lesion of the corpus callosum with diffusion-weighted imaging, a finding previously reported in both SNCM [36] and CM [37]. Another patient with SNCM with a slight reduction in GCS score had cytotoxic edema at MR imaging, a common feature of CM not observed in UM. These imaging findings indicate pathophysiological overlap of brain alteration between SNCM and CM.

Cytotoxic edema can be caused by hypoxia of the brain related to the compromised microcirculation in severe malaria. Contributing factors include IE sequestration, reduced erythrocyte deformability, adherence of IEs to uninfected erythrocytes (rosetting), and endothelial dysfunction [38]. In the current study, high parasite biomass was associated with both low and high ADC values in patients with SNCM. We thus explored whether additional factors were correlated with cytotoxic or vascular edema in this patient category.

While there were no significant associations between bilirubin or hemoglobin levels and ADC values, high creatinine levels were correlated with decreased whole-brain ADC values in patients with SNCM, suggesting a contribution of AKI to cytotoxic brain damage. Remarkably, the classic MR imaging pattern associated with AKI-induced uremic encephalopathy, characterized by increased ADC values in the basal ganglia, was not observed. In contrast, we detected a global decrease in whole-brain ADC values, with or without ADC decrease in the basal ganglia in patients with SNCM and high creatinine values. This imaging pattern is redolent of the findings we previously described in adult CM [14], and it suggests that impaired kidney function causes reduced cerebral blood flow, as has been shown in other conditions [39].

Further factors not assessed in this study may also contribute to cytotoxic brain edema. There is mounting evidence for the presence of kidney-brain cross talk in patients with AKI, leading to both direct and indirect cerebral insults [40]. Indeed, the central nervous system is vulnerable during AKI and chronic kidney disease [41], and potential contributing factors for brain involvement after AKI include the retention of nitrogenous end products (uremic toxins), osmolality disturbance, and inflammatory mechanisms, with resultant neutrophil migration, cytokine production, and increased oxidative stress [42]. While AKI is a well-established complication of severe malaria in Asian adults [43], it has been identified as common in African pediatric cohorts. In the latter age group, AKI is associated with mortality risk [44] and, more remarkably, with short- and long-term impaired cognition in survivors [45]. Indeed, AKI has been linked to hippocampus inflammation, cytotoxicity, and apoptosis, resulting in long-term cognitive impairment [19]. Studies investigating the association between AKI, brain changes at MR imaging, and neurocognitive outcomes are currently lacking in adults with severe malaria.

Collectively, our findings indicate a wide spectrum of pathological changes in the brain during severe and nonsevere falciparum malaria, despite the lack of deep coma. Not all patients with SNCM showed complete reversal of ADC changes during hospitalization. These observations may explain the development of postmalaria neurological syndrome, a rare self-limiting neurological syndrome after CM, but also sporadically after SNCM, is characterized by various neuropsychiatric manifestations ranging from mild neurological deficit to severe encephalopathy [46, 47].

Our study has limitations. First, logistical issues prevented us from using the Kidney Disease: Improving Global Outcomes guidelines to define AKI and stage kidney injury severity, which could have helped identify a broader range of kidney function impairment in our cohort [38, 44]. Correlations between ADC values and hyperlactatemia were not investigated owing to missing data points. Because both hypoxic lesions [48] and mild neurocognitive impairment [49] can be associated with hyperlactatemia, further analyses are needed to evaluate the effect of lactic acidosis on brain changes and long-term effects in SNCM. Finally, differences in retinopathy patterns between the high- and low-ADC groups were not assessed but are currently underway.

In conclusion, brain involvement is common in adults with SNCM and a subgroup of hospitalized patients with UM. Our findings suggest that severe malaria leads to a spectrum of neurological findings, where CM is only defined by the presence of coma. AKI in patients with SNCM may render the brain more susceptible to local hypoxia induced by parasite sequestration and resultant cytotoxic edema, leading to MR imaging features seen in CM. Additional studies aimed at investigating potential long-term neurocognitive deficits in patients with SNCM are warranted, and the need for a change in diagnostic criteria to facilitate the identification of patients for neurological follow-up, rehabilitation, and recovery must be considered in the future.

## Supplementary Data

Supplementary materials are available at *Clinical Infectious Diseases* online. Consisting of data provided by the authors to benefit the reader, the posted materials are not copyedited and are the sole responsibility of the authors, so questions or comments should be addressed to the corresponding author.

ciab907_suppl_Supplementary_Material

## Notes

***Acknowledgments.*** The authors thank the patients and their guardians/families for their participation in this study, as well as Nakul Chandra Khatua and Tapas Kar, the magnetic resonance imaging technicians at Ispat General Hospital, for their enthusiasm and logistical support. We acknowledge Saroj Mishra (deceased) and Frederik Barkhof for their invaluable help during the setup of the project, the director in charge and the clinical staff of Ispat General Hospital in Rourkela for their support and dedication, and the director of the Institute of Life Sciences in Bhubaneswar for allowing them to use its Infectious Disease Biology Unit to conduct laboratory work in Rourkela.

***Financial support.*** This work was supported by the National Institute of Allergy and Infectious Diseases, National Institutes of Health (grants U19AI089676 to S. Mohanty and S. C. W. and R21AI142472 to A. H. and S. C. W.), the UK Medical Research Council (award MR/S009450/1 to S. C. W.), and the Wellcome Trust (Institutional Translational Partnership award 214227/Z/18/Z to S. C. W.).

***Potential conflicts of interest.*** M. B. reports receiving support, personal consulting fees, and personal fees for lectures, presentations, speakers bureaus, manuscript writing, or educational events from the German Research Foundation (Deutsche Forschungsgemeinschaft [DFG]), the European Union, Novartis, the Hopp Foundation, Eurostars, and Stryker. A. H. reports receiving a research grant from Guerbet. All other authors report no potential conflicts. All authors have submitted the ICMJE Form for Disclosure of Potential Conflicts of Interest. Conflicts that the editors consider relevant to the content of the manuscript have been disclosed.
