# Supplementary material for: Evidence of Brain Alterations in Noncerebral Falciparum Malaria
Source: Clin Infect Dis. 2021 Dec 15;75(1):11–8. doi: 10.1093/cid/ciab907 (PMC9402700; doi:10.1093/cid/ciab907)
Supplement: ciab907_suppl_Supplementary_Material [file ciab907_suppl_supplementary_material.pdf]

SUPPLEMENTAL MATERIAL

Patients and Methods

1. *Ethical approvals*

Ethical approval was obtained from the Indian Council of Medical Research (TDR589/2010/ECDII), IGH, New York University School of Medicine (S12-03016), the London School of Hygiene and Tropical Medicine, and Heidelberg University Hospital.

2. *Patients and clinical definitions*

This study focused on adult patients (>16 years) with UM or SNCM. We were unable to include a pediatric group, owing to the challenges associated with performing a 40-minute MRI scan on febrile, non-comatose children. Falciparum malaria infections were confirmed by *P. falciparum* histidine-rich protein II antigen detection-based rapid diagnostic tests (SD Bioline, Standard Diagnostics, India), and subsequently standard microscopic examinations of Giemsa-stained thick and thin smears were carried out to corroborate species identification and assess parasitemia. SNCM patients were defined as having one or more severe malaria criteria [1], with the exception of coma (GCS ≥11 out of 15). These included *(i)* severe malarial anemia (Hb <7 g/dL), *(ii)* jaundice (bilirubin >3 mg/dL), and *(iii)* AKI (serum creatinine >3 mg/dL). UM was defined as slide-confirmed falciparum malaria with fever (axillary temperature, ≥37.5°C) or a history of fever in the preceding 24h, and absence of any severe malaria criterion. Additional exclusion criteria are detailed elsewhere [2]. All UM patients were hospitalized.

3. *Study procedures and clinical care*

On admission, a full medical history and physical examination were conducted and recorded on a standardised clinical record form. Blood samples were collected for cell and parasite counts, as well as biochemistry. Antimalarial treatment was with parenteral artesunate, provided in accordance with the national drug policy of the Government of India. Patient management followed the WHO recommendations [1].

4. <u>Brain swelling gradation & ADC analyses</u>

The degree of brain swelling was defined as: no brain swelling (grade 0); mild (grade 1: preserved sulci despite mild cortical swelling); moderate (grade 2: narrowing of adjacent cerebrospinal fluid-filled sulci); or severe brain swelling (grade 3: complete sulcal effacement). Grading was performed blindly by a board-certified neuroradiologist (AH) by generating a numbered image stack of all T2w datasets. ADC assessments were performed blindly by an experienced MRI post-processing image analysis expert (LP).

<u>Plasma level evaluation of S100B and PfHRP2</u>

Whole blood specimens (4.5 mL) were collected in sodium citrate tubes upon admission for all patients. Specimens were then immediately centrifuged at 500 *x g* for 10 minutes and plasma aliquots stored at -80°C prior to batch analyses. S100B, was quantified using a multiplexed bead-based ELISA (Luminex Corp, Austin, USA) and commercially available customizable kits (Human Magnetic Luminex Assay, R&D Systems, Minneapolis, USA). Plasma levels of *Plasmodium falciparum* histidine-rich protein 2 (PfHRP2), were assessed using commercially available ELISA kits (CellLabs, Sydney, Australia). All assays were performed according to the manufacturer protocols, in duplicate with results averaged for analyses, and by individuals blinded to study endpoints.

References:

1. WHO. Severe malaria. Trop Med Int Health **2014**; 19: 7-131.
2. Mohanty S, Benjamin LA, Majhi M, et al. Magnetic Resonance Imaging of Cerebral Malaria Patients Reveals Distinct Pathogenetic Processes in Different Parts of the Brain. mSphere **2017**; 2(3).

Supplemental Figure S1

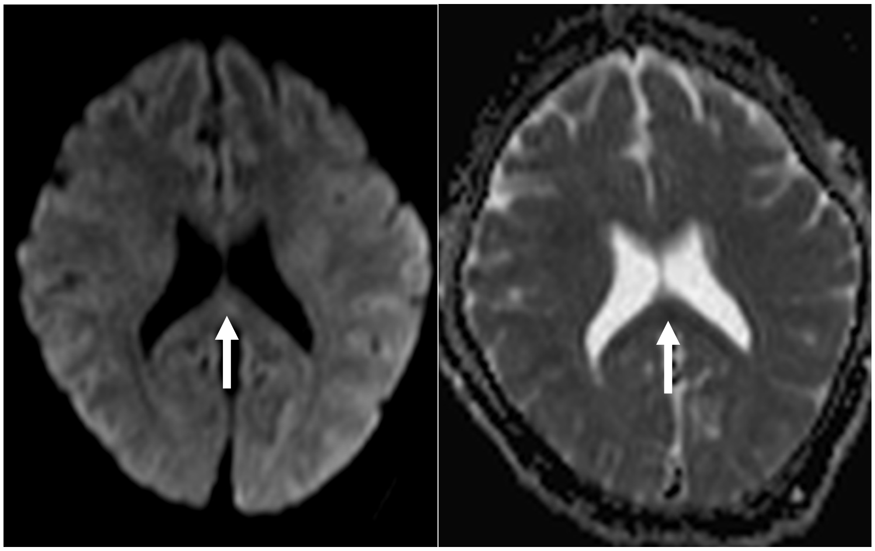

Cytotoxic lesion of the corpus callosum in an SNCM patient. A B1000 image and the corresponding ADC map with a small cytotoxic lesion of the corpus callosum (white arrows) are shown.

Supplemental Figure S2

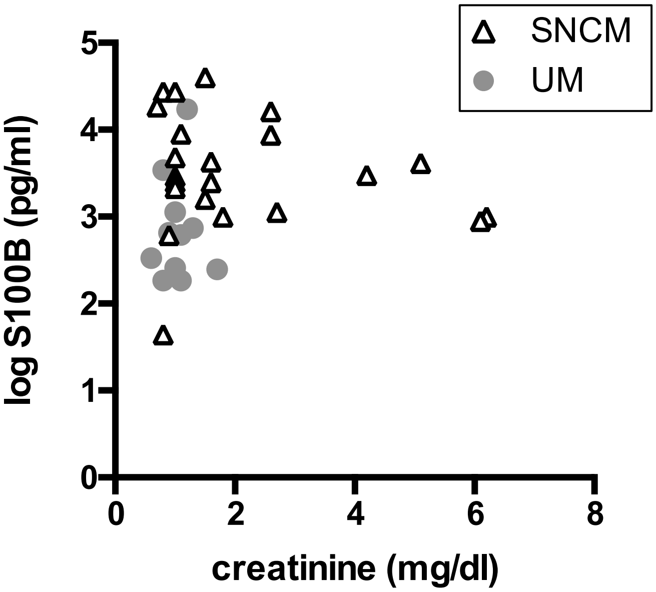

Plasma levels of S100B do not correlate with creatinine. Plasma levels of S100B are plotted against those of creatinine for both UM and SNCM patients.

Supplemental Figure S3

A

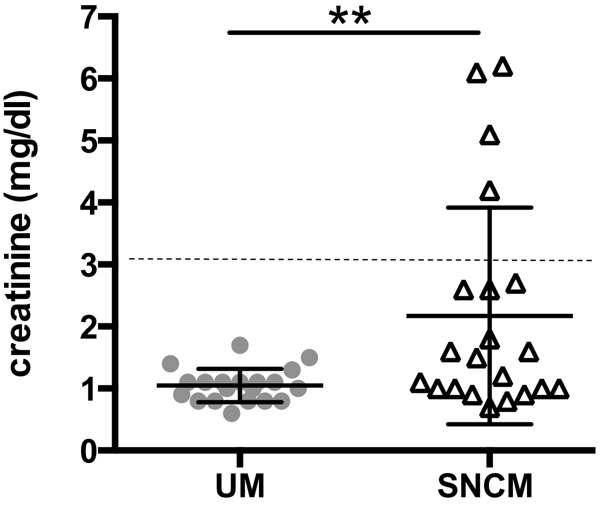

Laboratory values and ADC correlations in UM and SNCM patients. Box plots of creatinine (A), bilirubin (B, left) and hemoglobin (C, left) in UM and SNCM patients are presented. Black dotted lines present WHO cut-off values for jaundice and anemia in *Plasmodium* falciparum infection, respectively. Correlations of ADC values with bilirubin (B, right) and hemoglobin (C, right) are shown for UM and SNCM patients; the red line represent the lowest and the blue line the highest healthy control ADC value.

B

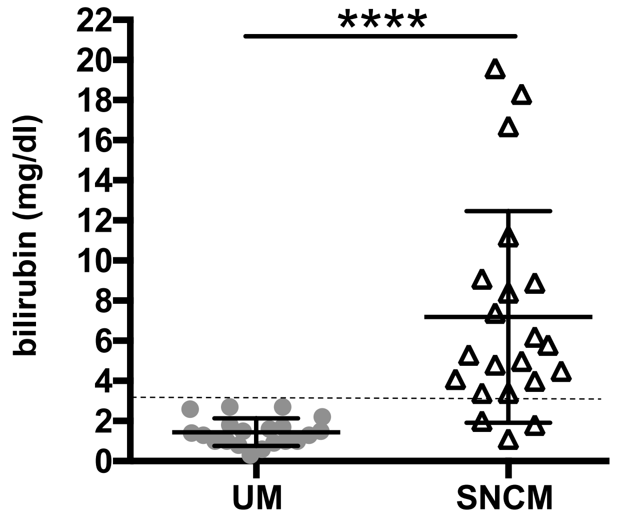

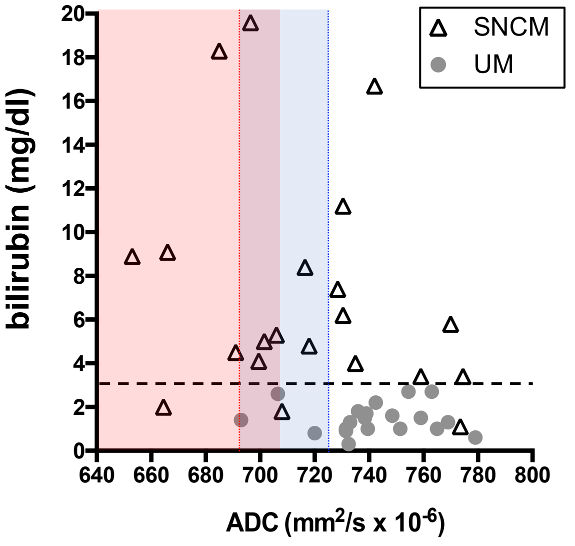

C

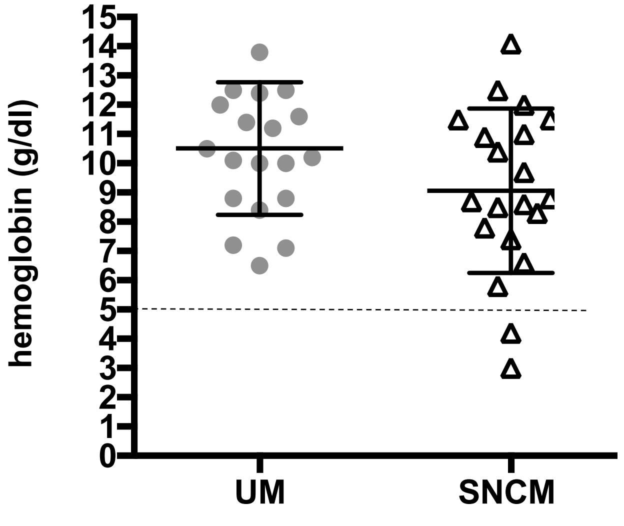

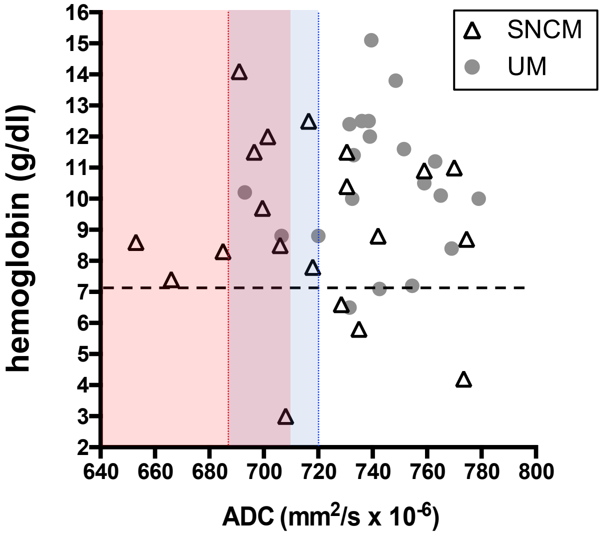

Supplemental Figure S4

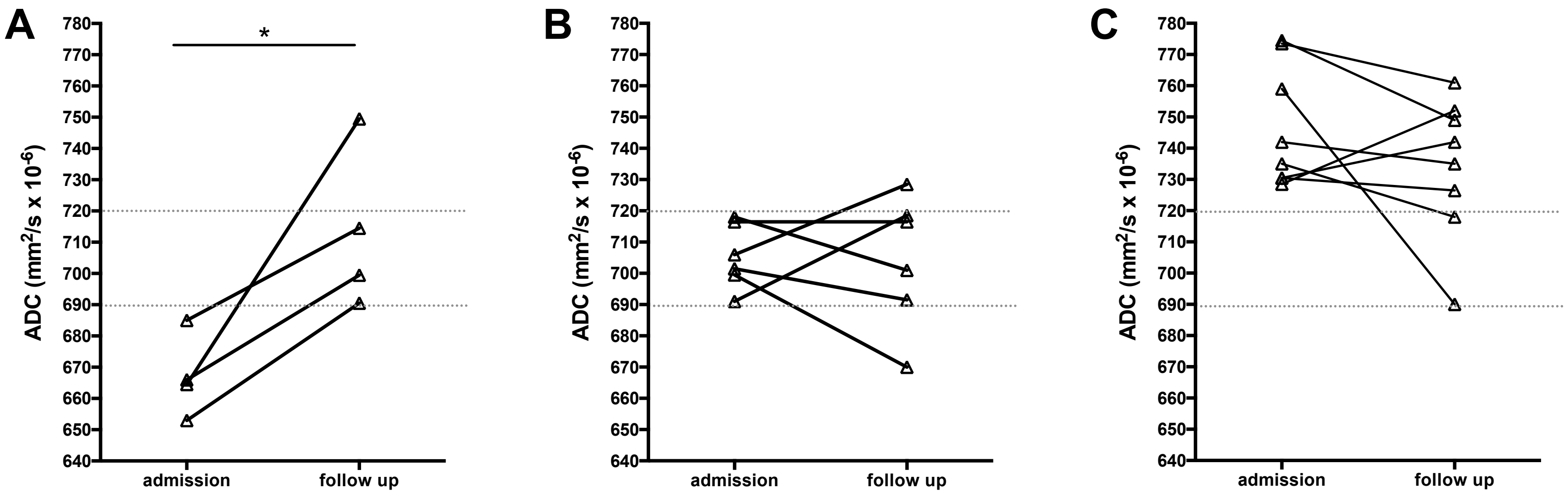

Temporal ADC changes in SNCM patients grouped according to ADC values on admission. ADC values on admission and follow-up are presented for SNCM patients who had low ADC values on admission compared to the healthy control range (A), ADC values on admission within the healthy control range (B), and ADC values on admission higher than the healthy control range (C).
